# Risk factors and clinical impact of prolonged air leak following video-assisted thoracoscopic surgery: a retrospective cohort study

**DOI:** 10.3389/fmed.2025.1549765

**Published:** 2025-03-03

**Authors:** Qingyun Ma, Evgeniy A. Tarabrin, Zelimkhan G. Berikkhanov, Milena Yu Ivanova

**Affiliations:** ^1^Department of Hospital Surgery No.2, I.M. Sechenov First Moscow State Medical University (Sechenov University), Moscow, Russia; ^2^National Medical Research Center of Pulmonology, I.M. Sechenov First Moscow State Medical University (Sechenov University), Moscow, Russia

**Keywords:** prolonged air leak (PAL), risk factors, complications, video-assisted thoracoscopic surgery (VATS), length of hospital stay

## Abstract

**Objective:**

This study aims to reveal the incidence and risk factors of prolonged air leak (PAL) following video-assisted thoracoscopic surgery (VATS) and to evaluate its impact on postoperative outcomes.

**Methods:**

A retrospective analysis was performed on the clinical data of all pulmonary surgery patients who underwent VATS at the Department of Hospital Surgery No.2 at Sechenov University, from September 2023 to September 2024. Patients were categorized into two groups based on the presence of PAL (defined as prolonged air leak lasting ≥ 5 days): the PAL group and the non-PAL group. Risk factors for PAL and its effects on postoperative recovery were assessed.

**Results:**

A total of 110 patients were included in the study, with an incidence of PAL of 26.3%. Multivariate analysis identified chronic obstructive pulmonary disease (COPD) (OR = 9.023, *P* = 0.003) and pleural adhesions (OR = 3.404, *P* = 0.013) as independent risk factors for the development of PAL. Significant differences were found between the PAL and non-PAL groups in terms of length of hospital stay (*P* < 0.001) and chest tube removal time (*P* < 0.001). The PAL group had a higher overall complication rate than the non-PAL group, with significantly more postoperative pneumonia (*P* = 0.003), postoperative empyema (*P* = 0.023), and postoperative wound infections (*P* = 0.005).

**Conclusion:**

Chronic obstructive pulmonary disease and pleural adhesions were identified as independent risk factors for PAL after VATS. Patients with PAL experienced more postoperative complications and longer hospital stays.

## Introduction

Video-assisted thoracic surgery (VATS) is a minimally invasive technique that has been widely applied in the diagnosis and treatment of pulmonary diseases according to various international guidelines (1–3). Compared to traditional open thoracotomy, VATS offers significant advantages, including smaller incisions, faster postoperative recovery, and improved visualization during surgery (4). However, prolonged air leakage (PAL) remains one of the most common and severe complications following VATS (5–9). PAL is defined as air leakage that persists for 5 days or longer after pulmonary surgery, leading to prolonged hospital stays, increased healthcare costs, and a range of postoperative complications, including atelectasis, pneumonia, wound infection, respiratory failure, and empyema (10, 11). Therefore, the aim of this study is to retrospectively analyze the incidence of PAL following VATS, identify associated risk factors, and explore its impact on postoperative outcomes, including hospitalization duration and complications.

## Materials and methods

The study was performed in accordance with the declaration of Helsinki and the subsequent revisions. The study was approved by the Local Ethical Committee of Sechenov University (#09-24 on 03.04.2024), and written informed consent was obtained from all patients.

### Patients

This study retrospectively analyzed the clinical data of patients who underwent VATS for pulmonary diseases including anatomical lung resection, pleurectomy, decortication, and atypical resections at the Department of Hospital Surgery No.2 at Sechenov University, from September 2023 to September 2024. A total of 110 patients were selected for the study.

The inclusion criteria were as follows:

1. Patients who underwent VATS for pulmonary surgery.
2. Patients who had not received preoperative radiotherapy or chemotherapy.
3. Patients aged 18 years or older.
4. Patients with complete clinical medical records.

The exclusion criteria were:

1. Patients who were converted from VATS to open thoracotomy during the operation.
2. Patients with preoperative infectious diseases or those with hematologic disorders, immune system diseases, or unexplained abnormal blood counts.
3. Patients who underwent VATS solely for diagnostic exploration or biopsy purposes.

### Clinical treatment protocol

All patients underwent VATS for pulmonary diseases, including anatomical lung resection, pleurectomy, decortication, and atypical resections. Preoperative evaluations indicated no absolute contraindications for surgery. The VATS procedures were performed one instrument port and one manual assistance port. Adhesions were taken down using a combination of blunt dissection and electrocautery to minimize tissue trauma. Bronchial and congenital fissures were routinely divided using a stapler. For malignant pulmonary lesions, lymph node sampling or systematic lymph node dissection was performed according to the guidelines of the National Comprehensive Cancer Network (NCCN) (12).

After completing the lung surgery, intraoperative air leaks were systematically assessed using a saline water test under sustained airway pressure (20 cmH2O). Air leaks were classified as follows: grade 1 (intermittent bubbles), grade 2 (continuous bubbles without tidal variation), and grade 3 (continuous bubbles with tidal variation or large leaks affecting ventilation). Only grade 3 leaks, defined as significant, were repaired with sutures. No biological adhesives or hemostatic agents were used during the procedure.

Postoperative passive chest tube drainage with no suction drainage system was performed. The chest tube air leakage was assessed twice daily by the appearance of air bubbles during breathing, talking and coughing under the water level in a bottle. Chest tubes were managed without suction unless clinically indicated, and all patients received routine pulmonary rehabilitation.

The criteria for chest tube removal were: no air leakage, drainage volume less than 200 ml over the previous 24 h, and imaging indicating satisfactory lung re-expansion.

### Data collection

This study systematically collected complete clinical data from patients, covering preoperative, intraoperative, and postoperative information. Preoperative data included the patients’ basic characteristics: age, gender, smoking history, body mass index (BMI), pulmonary function parameters, and major underlying conditions such as a history of diabetes, hypertension, and chronic obstructive pulmonary disease (COPD). The Modified Medical Research Council (mMRC) dyspnea score and Anesthesia and Surgical Assessment (ASA) score were also included to assess respiratory function and surgical tolerance.

Intraoperative data included detailed records of the type of surgery (anatomical lung resection, pleurectomy, pleural stripping, atypical resection, etc.), the surgical resection site, length of mechanical stapling, operative time, intraoperative blood loss, and the presence of thoracic adhesions.

Postoperative data focused on recovery within the first 9 days following surgery. Key data collected included the occurrence of pneumothorax, respiratory failure, and daily drainage volume during different time intervals (postoperative days 1–3, 4–5, 6–7, and 8–9), as well as the time of chest tube removal, the duration of air leakage, and major complications such as empyema, pneumonia, and wound infection. Total length of hospital stay was also recorded. Furthermore, laboratory test results were reviewed to obtain preoperative and postoperative inflammatory markers, including C-reactive protein (CRP), white blood cell count, and hemoglobin concentration.

### Statistical analysis

Data processing was performed using R statistical software (R4.2.3). A univariate analysis was conducted on the clinical data of patients in the PAL and non-PAL groups. The Kolmogorov–Smirnov test was used to assess the normality of continuous variables. Normally distributed variables were represented by means and standard deviations, while non-normally distributed variables were represented by medians and interquartile ranges (25th and 75th percentiles). Differences in normally distributed continuous variables were compared using the *t*-test, while differences in skewed distributions were compared using the Mann–Whitney *U* test. Intergroup comparisons were performed using the Chi-square test, with categorical data presented as *n* (%). A *P*-value of <0.05 was considered statistically significant.

## Results

### Patient demographic and clinical characteristics

A total of 110 patients (53 males and 57 females) were included in the study. The median age was 55.50 years (IQR: 42.00–64.75; overall range: 20–83), with a mean BMI of 26.15 kg/m^2^. A total of 12 patients (10.91%) were diagnosed with COPD. Forty-five patients (40.91%) had malignant tumors. The average operative time was 129.5 min, with an average intraoperative blood loss of 114 ml.

### Stratification of prolonged air leak

Based on the occurrence of PAL, the patients were divided into two groups, with patient characteristics summarized in Table 1. The incidence of PAL was 26.36% (29/110).

**TABLE 1:** Patient characteristics.

Characteristics	Total (*n* = 110)	Air leak < 5 days (*n* = 81)	Air leak ≥ 5 days (*n* = 29)	Statistic	*P*
Age, *M* (Q_1_, Q_3_)	55.50 (42.00, 64.75)	55.00 (41.00, 64.00)	58.00 (44.00, 67.00)	*Z* = 0.53	0.594
Gender, *n* (%)				χ^2^ = 0.20	0.656
Male	53 (48.18)	38 (46.91)	15 (51.72)		
Female	57 (51.82)	43 (53.09)	14 (48.28)		
BMI, *n* (%)				χ^2^ = 3.14	0.208
<18.5	7 (6.36)	7 (8.64)	0 (0.00)		
18.5–23.9	34 (30.91)	23 (28.40)	11 (37.93)		
≥24	69 (62.73)	51 (62.96)	18 (62.07)		
Smoking status, *n* (%)				χ^2^ = 2.42	0.120
Never smoked	70 (63.64)	55 (67.90)	15 (51.72)		
Current or former smoker	40 (36.36)	26 (32.10)	14 (48.28)		
COPD, *n* (%)				χ^2^ = 13.72	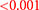
Without	98 (89.09)	78 (96.30)	20 (68.97)		
With	12 (10.91)	3 (3.70)	9 (31.03)		
Pleural adhesion, *n* (%)				χ^2^ = 10.27	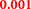
Without	62 (56.36)	53 (65.43)	9 (31.03)		
With	48 (43.64)	28 (34.57)	20 (68.97)		
FEV1, *n* (%)				–	0.106
<80%	14 (12.73)	7 (8.64)	7 (24.14)		
≥80%	16 (14.55)	12 (14.81)	4 (13.79)		
Unmeasured	80 (72.73)	62 (76.54)	18 (62.07)		
Diabetes, *n* (%)				χ^2^ = 0.00	0.961
Without	97 (88.18)	72 (88.89)	25 (86.21)		
With	13 (11.82)	9 (11.11)	4 (13.79)		
Malignant tumor, *n* (%)				χ^2^ = 0.25	0.617
Without	65 (59.09)	49 (60.49)	16 (55.17)		
With	45 (40.91)	32 (39.51)	13 (44.83)		
Respiratory failure, *n* (%)				χ^2^ = 3.00	0.083
Without	88 (80.00)	68 (83.95)	20 (68.97)		
With	22 (20.00)	13 (16.05)	9 (31.03)		
Hypertension, *n* (%)				χ^2^ = 0.99	0.321
Without	58 (52.73)	45 (55.56)	13 (44.83)		
With	52 (47.27)	36 (44.44)	16 (55.17)		
Preoperative hemoglobin, *M* (Q_1_, Q_3_)	129.00 (115.00, 139.00)	131.00 (114.75, 145.25)	124.00 (117.00, 135.00)	*Z* = 1.21	0.225
Preoperative leukocytes, *M* (Q_1_, Q_3_)	7.71 (5. 85, 12.56)	7.34 (5.62, 11.00)	9.52 (6.92, 14.74)	*Z* = 1.62	0.105
Preoperative CRP, *M* (Q_1_, Q_3_)	17.60 (3.00, 41.90)	13.80 (2.80, 44.50)	19.35 (3.88, 40.53)	*Z* = 0.01	0.991
Mechanical seam length, *M* (Q_1_, Q_3_)	180.00 (120.00, 180.00)	180.00 (120.00, 225.00)	180.00 (120.00, 180.00)	*Z* = 0.29	0.773
Duration of surgery, *M* (Q_1_, Q_3_)	120.00 (75.00, 165.00)	105.00 (65.00, 150.00)	150.00 (120.00, 195.00)	*Z* = 2.90	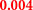
Blood loss, *M* (Q_1_, Q_3_)	30.00 (0.00, 137.50)	20.00 (0.00, 100.00)	100.00 (0.00, 300.00)	*Z* = 2.37	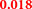
ASA I, *n* (%)				χ^2^ = 4.38	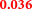
No	50 (45.45)	32 (39.51)	18 (62.07)		
Yes	60 (54.55)	49 (60.49)	11 (37.93)		
ASA II, *n* (%)				χ^2^ = 4.90	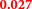
No	61 (55.45)	50 (61.73)	11 (37.93)		
Yes	49 (44.55)	31 (38.27)	18 (62.07)		
mMRC degree 0, *n* (%)				χ^2^ = 0.74	0.391
No	42 (38.18)	29 (35.80)	13 (44.83)		
Yes	68 (61.82)	52 (64.20)	16 (55.17)		
mMRC degree 1, *n* (%)				χ^2^ = 0.33	0.568
No	73 (66.36)	55 (67.90)	18 (62.07)		
Yes	37 (33.64)	26 (32.10)	11 (37.93)		
mMRC degree 2, *n* (%)				χ^2^ = 0.27	0.607
No	106 (96.36)	79 (97.53)	27 (93.10)		
Yes	4 (3.64)	2 (2.47)	2 (6.90)		
Anatomical resection, *n* (%)				χ^2^ = 2.13	0.144
No	62 (56.36)	49 (60.49)	13 (44.83)		
Yes	48 (43.64)	32 (39.51)	16 (55.17)		
Pleurectomy, *n* (%)				χ^2^ = 1.69	0.193
No	82 (74.55)	63 (77.78)	19 (65.52)		
Yes	28 (25.45)	18 (22.22)	10 (34.48)		
Decortication, *n* (%)				χ^2^ = 4.39	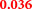
No	87 (79.09)	68 (83.95)	19 (65.52)		
Yes	23 (20.91)	13 (16.05)	10 (34.48)		
Atypical resection, *n* (%)				χ^2^ = 11.27	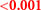
No	66 (60.00)	41 (50.62)	25 (86.21)		
Yes	44 (40.00)	40 (49.38)	4 (13.79)		
LUL, *n* (%)				χ^2^ = 0.67	0.415
No	67 (77.91)	54 (80.60)	13 (68.42)		
Yes	19 (22.09)	13 (19.40)	6 (31.58)		
LLL, *n* (%)				χ^2^ = 0.19	0.662
No	67 (77.91)	51 (76.12)	16 (84.21)		
Yes	19 (22.09)	16 (23.88)	3 (15.79)		
RUL, *n* (%)				χ^2^ = 0.03	0.867
No	69 (80.23)	53 (79.10)	16 (84.21)		
Yes	17 (19.77)	14 (20.90)	3 (15.79)		
ML, *n* (%)				χ^2^ = 0.00	0.965
No	79 (91.86)	61 (91.04)	18 (94.74)		
Yes	7 (8.14)	6 (8.96)	1 (5.26)		
RLL, *n* (%)				χ^2^ = 2.08	0.149
No	67 (77.91)	55 (82.09)	12 (63.16)		
Yes	19 (22.09)	12 (17.91)	7 (36.84)		
Number of drainage tubes, *n* (%)				χ^2^ = 7.91	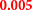
1	88 (80.00)	70 (86.42)	18 (62.07)		
2	22 (20.00)	11 (13.58)	11 (37.93)		

Bold values denote statistically significant results. No-PAL, no prolonged air leak; PAL, prolonged air leak; BMI, body mass index; COPD, chronic obstructive pulmonary disease; FEV1, forced expiratory volume in 1 s; ASA, Anesthesia and Surgical Assessment; mMRC, Modified Medical Research Council; LUL, left upper lobe; LLL, left lower lobe; RUL, right upper lobe; ML, middle lobe; RLL, right lower lobe.

### Surgical procedure distribution

The distribution of surgical procedures varied significantly between the two groups, as shown in Table 1. The PAL group (≥5 days) demonstrated a higher proportion of decortications (34.5% vs. 16.0%, *P* = 0.036) but lower rate of atypical resections (13.8% vs. 49.4%, *P* < 0.001). In contrast, the distributions of anatomical resections (55.2% vs. 39.5%, *P* = 0.144) and pleurectomies (34.5% vs. 22.2%, *P* = 0.193) showed no statistically significant differences.

### Risk factors for PAL

Univariate analysis revealed significant differences between groups in terms of COPD (*P* < 0.001), thoracic adhesions (*P* = 0.001), operative time (*P* = 0.004), intraoperative blood loss (*P* = 0.018), ASA I (*P* = 0.036), ASA II (*P* = 0.027), pleurectomy (*P* = 0.036), and atypical resection (*P* < 0.001), all of which were statistically significant (Table 1).

Further multivariate analysis confirmed that the presence of COPD (OR = 9.023, *P* = 0.003) and thoracic adhesions (OR = 3.404, *P* = 0.013) were independent risk factors for the development of PAL (Table 2).

**TABLE 2:** Results of logistic regression analysis.

	β	SE	Wals	*P*	OR	95% CI
COPD	2.200	0.737	8.914	0.003	9.023	2.129–38.240
Pleural adhesion	1.225	0.491	6.219	0.013	3.404	1.300–8.914
Intercept	1.955	0.382	26.232	0.000	0.142	

β, regression coefficient; SE, standard error; Wals, Wald statistic; *P*, *P*-value; OR, odds ratio; 95% CI, 95% confidence interval; COPD, chronic obstructive pulmonary disease.

### Impact of PAL on postoperative outcomes

Analysis of postoperative outcomes showed significant differences between patients in the non-PAL group and the PAL group. The PAL group experienced a significantly higher incidence of air leak, pneumothorax, and respiratory failure across all postoperative periods (days 1–3, 4–5, 6–7, and 8–9) compared to the non-PAL group. The drainage volume in the PAL group was significantly higher than that in the non-PAL group at all time points, with differences being statistically significant (*P* < 0.001).

Moreover, the PAL group had significantly lower hemoglobin levels on postoperative days 1–3 and 4–5 (*P* < 0.05), though no significant differences were observed in white blood cell count or CRP levels. The incidence of postoperative complications was higher in the PAL group, including empyema (*P* = 0.023), pneumonia (*P* = 0.003), and wound infection (*P* = 0.005) (Table 3).

**TABLE 3:** Impact of PAL on postoperative outcomes.

Variables	Total (*n* = 110)	Non-PAL group (*n* = 81)	PAL group (*n* = 29)	Statistic	*P*
POD 1–3 air leak, *n* (%)				χ^2^ = 59.07	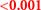
Without	66 (60.00)	66 (81.48)	0 (0.00)		
With	44 (40.00)	15 (18.52)	29 (100.00)		
POD 1–3 pneumothorax, *n* (%)				χ^2^ = 19.95	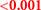
Without	83 (75.45)	70 (86.42)	13 (44.83)		
With	27 (24.55)	11 (13.58)	16 (55.17)		
POD 1–3 respiratory failure, *n* (%)				χ^2^ = 6.04	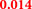
Without	89 (80.91)	70 (86.42)	19 (65.52)		
With	21 (19.09)	11 (13.58)	10 (34.48)		
POD 1–3 drainage fluid volume, *M* (Q_1_, Q_3_)	150.00 (100.00, 300.00)	100.00 (50.00, 200.00)	300.00 (200.00, 400.00)	*Z* = 5.19	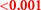
POD 1–3 hemoglobin, *M* (Q_1_, Q_3_)	121.00 (106.00, 133.00)	123.50 (109.75, 135.25)	111.00 (98.00, 121.00)	*Z* = 2.98	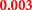
POD 1–3 leukocytes, *M* (Q_1_, Q_3_)	10.10 (7.63, 11.99)	10.18 (7.43, 11.88)	9.84 (8.61, 12.75)	*Z* = 0.52	0.600
POD 1–3 C-reactive protein, *M* (Q_1_, Q_3_)	51.20 (25.00, 109.30)	45.30 (22.88, 92.25)	79.50 (35.50, 155.10)	*Z* = 1.50	0.134
POD 4–5 air leak, *n* (%)				χ^2^ = 110.00	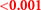
Without	81 (73.64)	81 (100.00)	0 (0.00)		
With	29 (26.36)	0 (0.00)	29 (100.00)		
POD 4–5 pneumothorax, *n* (%)				χ^2^ = 23.61	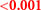
Without	85 (77.27)	72 (88.89)	13 (44.83)		
With	25 (22.73)	9 (11.11)	16 (55.17)		
POD 4–5 respiratory failure, *n* (%)				χ^2^ = 2.58	0.108
Without	95 (86.36)	73 (90.12)	22 (75.86)		
With	15 (13.64)	8 (9.88)	7 (24.14)		
POD 4–5 drainage fluid volume, *M* (Q_1_, Q_3_)	50.00 (0.00, 150.00)	0.00 (0.00, 100.00)	150.00 (100.00, 200.00)	*Z* = 5.89	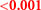
POD 4–5 hemoglobin, *M* (Q_1_, Q_3_)	115.50 (103.50, 127.25)	117.00 (108.50, 136.00)	109.00 (101.50, 120.50)	*Z* = 2.28	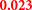
POD 4–5 leukocytes, *M* (Q_1_, Q_3_)	8.25 (6.18, 11.02)	8.96 (6.17, 11.35)	7.77 (6.24, 10.57)	*Z* = 0.53	0.593
POD 4–5 C-reactive protein, *M* (Q_1_, Q_3_)	46.90 (19.68, 100.35)	50.95 (19.50, 107.10)	45.80 (25.20, 80.45)	*Z* = 0.27	0.785
POD 6–7 air leak, *n* (%)				χ^2^ = 55.66	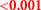
Without	92 (83.64)	81 (100.00)	11 (37.93)		
With	18 (16.36)	0 (0.00)	18 (62.07)		
POD 6–7 pneumothorax, *n* (%)				χ^2^ = 36.22	**<0.001**
Without	90 (81.82)	77 (95.06)	13 (44.83)		
With	20 (18.18)	4 (4.94)	16 (55.17)		
POD 6–7 respiratory failure, *n* (%)				χ^2^ = 6.10	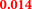
Without	101 (91.82)	78 (96.30)	23 (79.31)		
With	9 (8.18)	3 (3.70)	6 (20.69)		
POD 6–7 drainage fluid volume, *M* (Q_1_, Q_3_)	0.00 (0.00, 95.00)	0.00 (0.00, 20.00)	100.00 (50.00, 150.00)	*Z* = 5.52	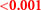
POD 6–7 hemoglobin, *M* (Q_1_, Q_3_)	113.00 (99.75, 128.25)	113.00 (102.00, 132.00)	107.00 (98.00, 125.00)	*Z* = 0.90	0.369
POD 6–7 leukocytes, *M* (Q_1_, Q_3_)	8.14 (6.08, 10.38)	8.35 (6.07, 10.07)	7.78 (6.43, 10.33)	*Z* = 0.13	0.895
POD 6–7 C-reactive protein, *M* (Q_1_, Q_3_)	34.80 (18.65, 95.75)	39.50 (16.25, 100.35)	34.80 (23.55, 69.45)	*Z* = 0.03	0.974
POD 8–9 air leak, *n* (%)				χ^2^ = 17.03	**<0.001**
Without	103 (93.64)	81 (100.00)	22 (75.86)		
With	7 (6.36)	0 (0.00)	7 (24.14)		
POD 8–9 pneumothorax, *n* (%)				χ^2^ = 7.98	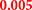
Without	102 (92.73)	79 (97.53)	23 (79.31)		
With	8 (7.27)	2 (2.47)	6 (20.69)		
POD 8–9 respiratory failure, *n* (%)				χ^2^ = 1.51	0.220
Without	105 (95.45)	79 (97.53)	26 (89.66)		
With	5 (4.55)	2 (2.47)	3 (10.34)		
POD 8–9 drainage fluid volume, *M* (Q_1_, Q_3_)	0.00 (0.00, 0.00)	0.00 (0.00, 0.00)	50.00 (0.00, 70.00)	*Z* = 5.21	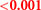
POD 8–9 hemoglobin, *M* (Q_1_, Q_3_)	115.00 (105.00, 126.50)	116.50 (104.75, 130.75)	113.00 (106.50, 123.00)	*Z* = 0.88	0.379
POD 8–9 leukocytes, *M* (Q_1_, Q_3_)	8.31 (6.55, 9.43)	8.30 (6.20, 9.15)	8.33 (6.96, 9.43)	*Z* = 0.55	0.585
POD 8–9 C-reactive protein, *M* (Q_1_, Q_3_)	25.20 (13.20, 60.90)	28.10 (15.22, 73.75)	25.20 (12.65, 55.30)	*Z* = 0.07	0.946
Postoperative empyema, *n* (%)				χ^2^ = 5.16	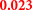
Without	88 (80.00)	69 (85.19)	19 (65.52)		
With	22 (20.00)	12 (14.81)	10 (34.48)		
Postoperative pneumonia, *n* (%)				χ^2^ = 9.01	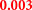
Without	74 (67.27)	61 (75.31)	13 (44.83)		
With	36 (32.73)	20 (24.69)	16 (55.17)		
Postoperative wound infection, *n* (%)				χ^2^ = 7.73	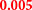
Without	104 (94.55)	80 (98.77)	24 (82.76)		
With	6 (5.45)	1 (1.23)	5 (17.24)		
Removal of drains, *M* (Q_1_, Q_3_)	5.50 (3.00, 8.00)	4.00 (2.00, 6.00)	9.00 (7.00, 13.00)	*Z* = 5.93	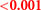
Duration hospitalization, *M* (Q_1_, Q_3_)	14.00 (9.00, 17.75)	12.00 (9.00, 17.00)	16.00 (15.00, 21.00)	*Z* = 4.17	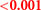

Bold values denote statistically significant results. No-PAL, no prolonged air leak; PAL, prolonged air leak; POD, postoperative day.

**FIGURE 1:**
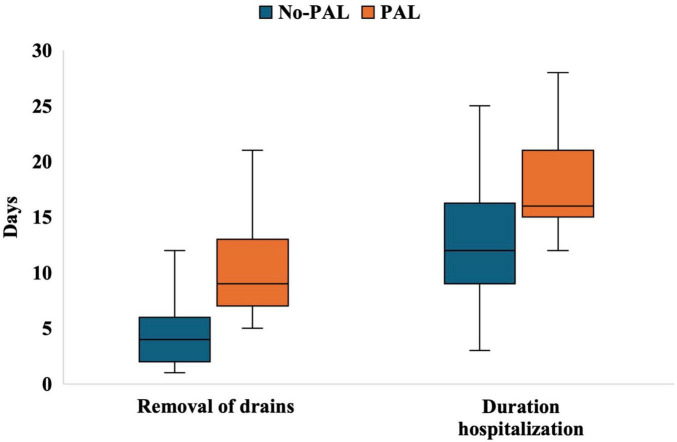
Boxplots of chest tube removal time and length of hospital stay in the non-PAL and PAL groups. No-PAL, no prolonged air leak; PAL, prolonged air leak.

Additionally, patients in the PAL group had a significantly longer time to chest tube removal (9 days vs. 4 days, *P* < 0.001) and a longer length of hospital stay (16 days vs. 12 days, *P* < 0.001) (Figure 1).

## Discussion

Prolonged air leak is one of the most common complications following VATS, significantly affecting patients’ postoperative clinical recovery. The mechanism underlying the development of PAL may be associated with alveolopleural fistula or bronchopleural fistula, which are pathological changes that allow air to escape from the lung tissue or bronchi into the pleural cavity (13, 14). According to previous literature, the incidence of PAL typically ranges from 5% to 26% (10, 11, 15–17). In this study, the incidence of PAL was 26.3%, which exceeds the current reported range. This may be related to the presence of significant preoperative comorbidities, the high incidence of COPD (10.9%), and the specific regional and demographic characteristics of the study population. Additionally, this result could be influenced by the complexity of the surgery and the intraoperative techniques employed.

While the observed high percentage of COPD in our population may suggest that COPD is a major risk factor for PAL, it is important to consider whether this high prevalence could bias the results. The relatively large proportion of COPD patients in our study (10.9%) may have disproportionately increased the observed effect size of COPD as a risk factor for PAL. This raises the question of whether the association we observed between COPD and PAL is a true reflection of the risk, or if it is influenced by the overrepresentation of COPD patients in the sample.

To address this potential bias, future studies could consider stratifying the sample based on COPD status or conducting sensitivity analyses to assess the robustness of COPD as an independent risk factor for PAL in populations with different baseline prevalences of COPD. Nevertheless, our multivariate regression analysis still supports a significant association between COPD and PAL (OR = 9.023, *P* = 0.003), consistent with findings from previous studies (18, 19). Notably, a study by Meacci et al. (20) also identified COPD as a risk factor for PAL, despite the fact that 28 of their patients (22.9%) had COPD. This suggests that even when COPD prevalence is higher, its role as a risk factor remains significant. Therefore, our findings align with previous research, reinforcing the importance of COPD as a contributing factor to PAL development.

Chronic obstructive pulmonary disease is characterized by the destruction of elastic fibers and thinning of the alveolar walls, which significantly increases the risk of alveolar rupture and air leak. Preoperative respiratory function impairment (such as reduced FEV1) in COPD patients may further compromise the lung’s ability to repair itself postoperatively. A study by Ponholzer et al. (21) indicated that PAL was associated with preoperative COPD diagnosis (52.4% vs. 30.1%, *P* < 0.001). Among all COPD patients, 17.4% experienced PAL (vs. 7.6%, *P* < 0.001), and FEV1 as a percentage of predicted values was lower in COPD patients (74.4% vs. 82.5%, *P* < 0.001) (21).

Additionally, pleural adhesions were identified as another independent risk factor for PAL (OR = 3.404, *P* = 0.013), consistent with previous literature (22). Pleural adhesions not only increase the technical difficulty of separating tissues during surgery but may also cause local tissue damage due to mechanical separation, further increasing the risk of PAL. Zheng et al. (23), through a meta-analysis, found that nearly 17 studies demonstrated that pleural adhesions significantly increased the risk of PAL (*P* < 0.001), and the severity of pleural adhesions was correlated with higher rates of intraoperative air leaks.

These findings highlight the importance of preoperative assessment and perioperative management strategies to mitigate the risk of PAL, particularly in patients with COPD and pleural adhesions. Future studies should further explore interventions that could improve postoperative outcomes in these high-risk populations.

In addition to COPD and pleural adhesions, our study highlights the significant impact of surgical factors on PAL development. We found that operative time (*P* = 0.004) and blood loss (*P* = 0.018) in the PAL group were significantly greater than those in the non-PAL group. Prolonged operative time may be associated with increased surgical complexity and prolonged exposure of lung tissue during the procedure. Previous studies have indicated that for every 30-min increase in surgical time, the risk of postoperative complications increases by approximately 14% (24). Significant intraoperative blood loss may lead to local ischemia of lung tissue, delaying the healing of air leaks. Increased blood loss may also exacerbate postoperative inflammatory responses, further contributing to the development of PAL.

Prolonged air leak has a significant negative impact on postoperative recovery. Patients in the PAL group had a significantly longer time to chest tube removal (median 9 days vs. 4 days) and a longer hospital stay (median 16 days vs. 12 days). Konstantinidis et al. (25), in a retrospective study, found that the 90-day readmission rate for PAL patients was 24%, significantly higher than the 9.2% for non-PAL patients (35/380, *P* < 0.0001).

In this study, the incidence of postoperative complications, including pneumonia, empyema, and wound infection, was significantly higher in the PAL group compared to the non-PAL group. This may be related to inadequate intrathoracic negative pressure and poor lung re-expansion. In addition, PAL may further trigger secondary complications such as pneumothorax and pleural effusion. Moreover, PAL also significantly increases the risk of respiratory failure, severely threatening postoperative recovery. Although no significant differences were found between the PAL and non-PAL groups in terms of white blood cell count and CRP levels, the PAL group had significantly lower hemoglobin levels postoperatively. This may indicate chronic blood loss or excessive drainage, leading to anemia, which could further weaken the immune function and repair capacity during the postoperative recovery period.

This study is a single-center retrospective design with a relatively small sample size, and the follow-up data of FEV1 were incomplete. Future prospective multi-center study with larger sample size should be conducted to validate the risk factors for PAL and the impact of PAL on the postoperative outcomes. In addition, as the advancement of bioengineering and biomaterials, the potential application of novel intraoperative techniques in the prevention of PAL should be prioritized for improved prognosis.

## Conclusion

This study analyzed the incidence, risk factors, and impact of PAL on postoperative outcomes in patients undergoing VATS. The study found that COPD and pleural adhesions are independent risk factors for PAL. The occurrence of PAL significantly prolongs hospital stays, increases the incidence of postoperative complications, and negatively affects patients’ postoperative recovery and quality of life. Future efforts should focus on optimizing preoperative assessment and intraoperative techniques to develop personalized intervention strategies, aiming to reduce the incidence of PAL and improve postoperative outcomes.

## Data Availability

The raw data supporting the conclusions of this article will be made available by the authors, without undue reservation.
